# A chemodosimeter-modified carbon nanotube-field effect transistor: toward a highly selective and sensitive electrical sensing platform†

**DOI:** 10.1039/c9ra04656a

**Published:** 2019-09-09

**Authors:** Chang-Seuk Lee, Jong Seung Kim, Tae Hyun Kim

**Affiliations:** Department of Chemistry, Soonchunhyang University Republic of Korea thkim@sch.ac.kr +82-41-530-4722; Department of Chemistry, Korea University Republic of Korea jonskim@korea.ac.kr +82-2-3290-3183

## Abstract

We present a carbon nanotube-field effect transistor (CNT-FET) biosensor which first implements the chemodosimeter sensing principle in CNT nanoelectronics. We experimentally illustrate the specific molecular interplay that the cysteine-selective chemodosimeter immobilized on the CNT surface can specifically interact with cysteine, which leads to the chemical transformation of the chemodosimeter. Since the chemical transformation of the chemodosimeter can disrupt the charge distribution in the vicinity of the CNT surface, the carrier equilibrium in CNT might be altered, and manifested by the conductivity change of CNT-FET. The real-time conductance measurements show our biosensor is capable of label-free, rapid, highly selective and ultrasensitive detection of cysteine with a detection limit down to 0.45 fM. These results first verify the signaling principle competency of chemical transformation of the chemodosimeter in CNT electronic sensors. Combined with the advantages of the highly selective chemodosimeter and sensitive CNT-FET, the excellent performance of our sensor indicates its promising prospect as a valuable tool for developing highly sensitive and selective sensing platforms in practical application.

## Abstract 2

The utility of the chemodosimetric sensing principle was demonstrated for the first time in electronic biosensing with CNT-FET devices.

## Graphical abstract

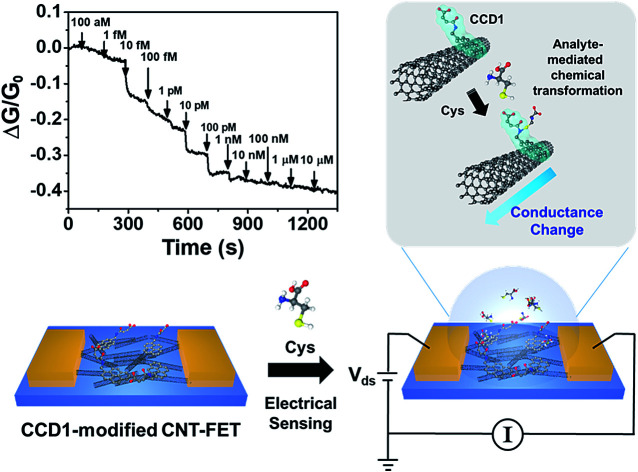

## 1. Introduction

Portable nanosensor systems that can perform sensitive, selective and real-time monitoring of analytes are imperative for disease diagnostics, food safety, and environmental monitoring.^1–3^ With the recent advent of nano- and biotechnology, sensor technologies have seen great progress in detecting various target analytes. Nanoelectronic devices such as carbon nanotube (CNT) based devices have demonstrated highly sensitive detection of chemical and biological species, which is owing to the environmentally-sensitive electronic properties of CNTs.^4–6^ In particular, the employment of CNTs in field-effect transistors (FETs) has attracted much attention in the development of sensor systems due to the potential of nanostructured devices as superior biosensing and diagnostics tools.^7–9^ However, despite intensive efforts devoted to the development of the CNT-FET sensing devices, they often suffer from a lack of selectivity such as the false positive responses against interferences. Therefore, current CNT-FET sensing systems are still far from realization to meet the requirements of selective, sensitive, and real-time point-detection. Various attempts have been made for inclusion of selective recognition units, such as bioreceptor and chemical probes, in CNT-FET devices.^10–14^ However, improvements are still needed to develop new technologies or materials to bridge sensitive CNT-FET devices and selective recognition units for the targets of interest with little loss of either sensitivity or selectivity. Here, we describe a novel sensor that first incorporates the chemodosimeter-sensing principle in a CNT nanoelectronic device. Chemodosimeters are used to achieve analyte recognition through a specific and irreversible chemical reaction involving both breaking and forming of the covalent bonds, which is relatively less affected by the environment.^15–18^ Compared with coordination-based chemosensors, chemodosimeters can provide excellent selectivity for the detection of analytes. In addition, chemodosimeters are advantageous over bioreceptors in terms of cost-effectiveness and rapid analysis. Analyte-mediated chemical transformation of the chemodosimeter may enable highly selective electrical sensing principle that transduces the specific chemical reaction into electrical signals *via* the charge redistribution on CNT-FET.

**Scheme 1:**
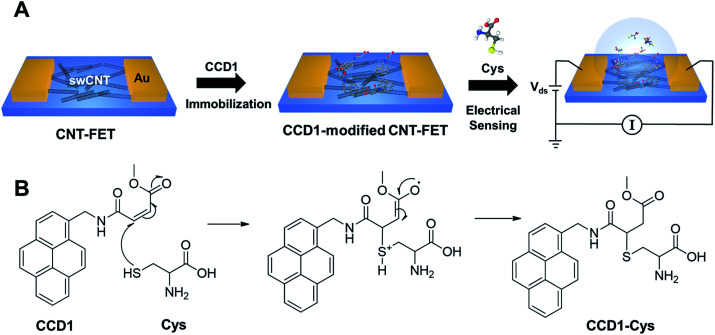
(A) Illustration of the fabrication of chemodosimeter (CCD1)-modified CNT-FET biosensor for Cys detection. (B) Chemodosimetric reaction of CCD1 with Cys.

Scheme 1 illustrates the preparation process of a chemodosimeter-modified CNT-FET sensor and the sensing strategy. As shown in Scheme 1A, we fabricated CNT-FETs, as reported previously.^11–14,19^ The CNT surfaces on the FET channels were then functionalized with chemodosimeter molecules *via* π–π stacking interaction between the terminal pyrenes of chemodosimeters and the CNT. As a proof of concept, here, we used the cysteine (Cys)-selective chemodosimeter (CCD1) in which the β carbon of the conjugated acrylate moiety can react with the thiol of Cys *via* a Michael-type addition reaction to produce the corresponding adduct, CCD1-Cys, leading to an observable signal due to the change in photoelectrical property (Scheme 1B). The chemodosimetric reaction between Cys and CCD1 on CNT-FET enables the CNT-FET to detect Cys selectively through the change of charge distribution on the CNT surface arising from the chemical transformation. The work provides a simple and versatile approach to develop highly selective, sensitive and rapid sensing platforms for biological/chemical sensing, diagnostics, and drug screening.

## 2. Experimental

### 2.1. Chemicals and materials

Cys, homocysteine (Hcy), glutathione (GSH), methionine (Met), tryptophane (Trp), phenylalanine (Phe), 1,2-dichlorobenzene, acetonitrile, phosphate buffer saline (PBS), and 1-pyrenebutanol were purchased from Sigma-Aldrich Co. (St. Louis, MO, USA). Single-walled CNTs (swCNTs) were purchased from Hanwha Nanotech Co. (Korea). All the reagents were analytical grade and were used without further purification. Deionized (DI) water was prepared in a Millipore water purification system (MilliQ, specific resistivity >18 MΩ cm, Millipore Korea, Co., Ltd.).

### 2.2. Synthesis of CCD1

The synthetic route to CCD1 is illustrated in Scheme S1.† Briefly, 1-aminomethyl pyrene was dissolved in dichloromethane and then added a solution of 2,5-furandione in dichloromethane. The mixture was filtered, and the solvent was removed under reduced pressure. CCD2 was then obtained by purification using column chromatography. CCD1 was synthesized by reacting CCD2 with methanol and toluenesulfonic acid (TsOH).

### 2.3. Fabrication of CNT-based FETs

The method for fabricating CNT-FETs were similar to those reported previously.^11–14,19^ Briefly, electric circuit for FET was patterned on SiO_2_ surface using photolithography followed by thermal evaporation of Au/Ti (30 nm/10 nm) and a lift-off process. Afterwards, AZ5214 photoresist was patterned again to form a swCNT channels. The length and width of channels were 30 μm and 20 μm, respectively. When the patterned substrate was dipped in swCNT suspensions (0.1 mg mL^−1^ 1,2-dichlorobenzene) for 5 s, swCNTs were selectively adsorbed and aligned on the bare SiO_2_ surface regions. Finally, contact electrodes were patterned using photolithography followed by thermal evaporation of Au/Pd (30 nm/10 nm) and a lift-off process.

### 2.4 Functionalization of CNT-FETs with CCD1

The CNT junctions on the FETs were modified with CCD1 to fabricate biosensors for Cys detection. The CNT-FET device was dipped into a 0.5 mM CCD1 solution in CH_3_CN at −20 °C for 1 h. After incubation, the resulting CNT-FET device was rinsed with DI water 3 times and dried with pure N_2_ (99.999%) gas.

### 2.5 Instrumentation and measurements

The electrical measurements of CNT-FETs were performed with HP 4145B (Agilent HP, US) semiconductor parameter analyser. For Cys sensing, the CCD1 modified CNT-FETs were placed in a pH 7.4 PBS solution, and the source-drain currents were measured under a 100 mV source-drain bias after adding Cys at different concentrations. Fluorescence and UV/vis absorption spectra were recorded with Shimadzu RF-5301PC and Shinco S-3100 spectrophotometers, respectively. Fourier transform infrared (FT-IR) spectra were performed with a NICOLETiS10 (Thermo scientific Korea Ltd.). Energy dispersive spectroscopy (EDS) data were taken on a JSM-6701F (JEOL, Ltd., Japan). X-ray photoelectron spectroscopy (XPS) spectra were recorded with a K-alpha (Thermo VG Scientific, USA).

## 3. Results and discussion

### 3.1. Chemodosimetric behavior of CCD1 toward Cys

**Fig. 1:**
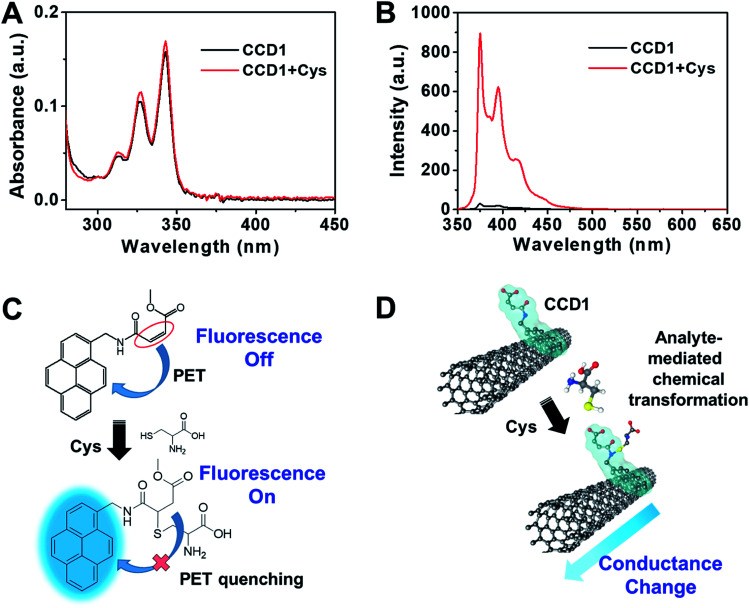
(A) UV-vis and (B) fluorescence spectra of CCD1 (5.0 μM) in aqueous solution (10 mM PBS buffer, pH 7.4, 10% DMSO) upon addition of Cys (100 equiv.). Excitation at 343 nm after 10 min (slit = 1.5/3). (C) Proposed chemodosimetric fluorescence sensing mechanism of CCD1 toward Cys. (D) Electrical response mechanism in CNT-FET based on the chemodosimetric reaction between CCD1 and Cys.

In order to ascertain the reactivity of CCD1 toward Cys, the photophysical properties of CCD1 were examined upon exposure to Cys by UV-vis and fluorescence spectroscopy. The absorption spectra of free CCD1 in aqueous solution (10 mM PBS buffer, pH 7.4, 10% DMSO) exhibited the characteristic absorption peaks at 313, 327 and 342 nm, which are similar to those of reported pyrene derivatives.^20^ In the presence of Cys, the absorption bands of CCD1 slightly increased (Fig. 1A). Interestingly, however, the emission spectra of CCD1 exhibited dramatic changes in the photophysical properties of CCD1 upon exposure to Cys (Fig. 1B). The probe CCD1 shows very weak emission at ∼375, 396 and 416 nm when excited at 343 nm in aqueous solution (10 mM PBS buffer, pH 7.4, 10% DMSO). Upon interaction with Cys, the fluorescence signal of CCD1 changed significantly. When 500 μM Cys were added to the solution of 5.0 μM CCD1, about 30-fold enhancement in fluorescence was observed at ∼376 nm. Meanwhile, free CCD2 bearing carboxyl group showed similar absorption and emission behaviors with CCD1, however, with the addition of Cys, CCD2 exhibited small change in fluorescence spectra (Fig. S1†). On the basis of the results from the fluorescence spectra, a possible mechanism of probe CCD1 and Cys was shown in Fig. 1C. CCD1 exhibited very weak fluorescence due to a photoinduced electron transfer (PET) reaction involving the pyrene moiety (fluorophore) and the nearby conjugated acrylate group.^21–24^ However, the original PET process in CCD1 was prohibited because the conjugation of the acrylate group was broken, during the Michael-type addition of Cys to CCD1, resulting in changes in fluorescence spectra. On the other hand, the Michael-type addition of Cys to CCD2 is somewhat difficult due to the carboxyl group of CCD2 which can be deprotonated to carboxylate ion in pH 7.4 buffer solution, making CCD2 hard to act as an electrophile. This change of the electric property arising from the chemodosimetric reaction can be designed to enable the electrical detection of Cys with CNT-FET (Fig. 1D). From the viewpoint of CNT-FET electronics, the analyte-mediated chemical transformation of CCD1 may alter the charge distribution on the swCNT channel in CCD1-modified CNT-FET, therefore, leading to the conductance change.

### 3.2. Characterization of the CCD1-modified CNT-FET

**Fig. 2:**
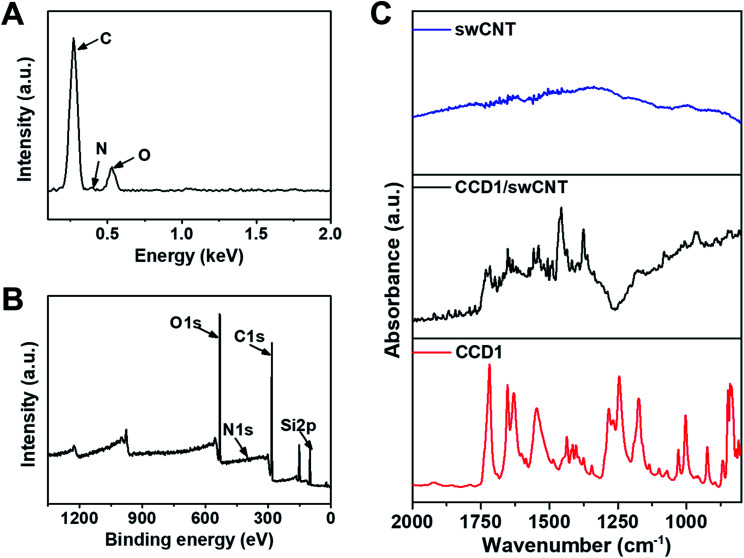
(A) EDS spectrum and (B) XPS full spectrum of CCD1-modified swCNTs. (C) FT-IR spectra of bare swCNTs, CCD1-modified swCNTs and CCD1.

Before the use of CCD1-modified CNT-FET as a Cys sensor, the characterization studies including EDS, XPS, and FT-IR experiments were conducted to confirm the functionalization of swCNTs with CCD1 molecules. The chemical composition was evaluated by EDS analysis to verify the presence of CCD1s on the swCNT surface. Fig. 2A shows the EDS profile of a CCD1-modified swCNT, where peaks of C and O, and weak peak of N were detected, which is due to the small quantity of N, compared with those of C and O. As shown in Fig. 2B, the XPS survey scan also revealed distinct C(1s), N(1s), and O(1s) peaks in a CCD1-modified swCNT. The appearance of the N(1s) and O(1s) peaks confirmed the existence of CCD1 with amine group and acrylate moiety, functionalized on the surface of swCNT. For the detail analysis of XPS spectra of CCD1-modified swCNTs show in ESI (Fig. S2†). FT-IR analysis was performed on the CCD1-modified swCNTs to investigate the presence of functional groups such as amine, carbonyl and ester groups of CCD1 on the swCNTs (Fig. 2C). Unlike that of bare swCNT, the FT-IR spectra of the swCNT after the modification of CCD1 exhibited two peaks at 1655 and 1250 cm^−1^, which are corresponding to the bending vibration mode of N–H and stretching mode C–N in amine group, respectively. In addition, peaks at 1740 and 1175 cm^−1^ are attributed to the stretching mode C=O and C–O in ester group. From all the results of EDS, XPS and FT-IR analysis, we can clearly confirm that CCD1 molecules were well modified on the surface of swCNTs.

### 3.3. Highly selective and ultrasensitive detection of Cys using CCD1-modified CNT-FET

**Fig. 3:**
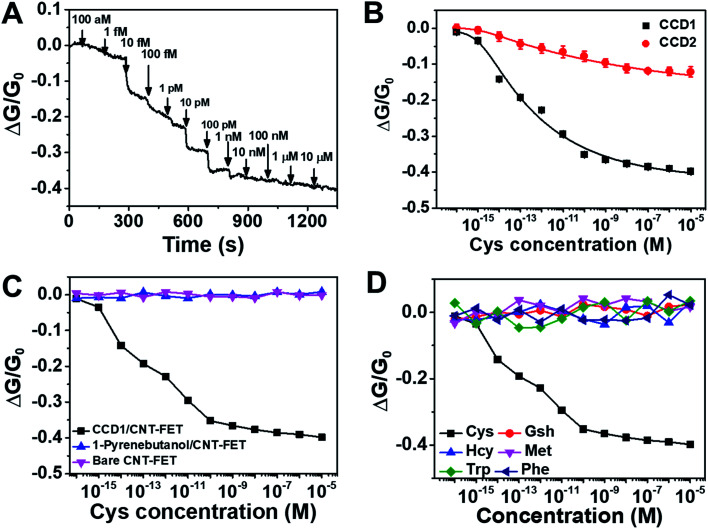
(A) Real-time conductance measurement data obtained from CCD1-modified CNT-FETs upon successive addition of Cys at various concentrations. Arrows indicate the injection points of Cys target molecules. (B) Calibration curve for Cys real-time conductance measurements obtained from the CCD1- and CCD2-modified CNT-FETs at various concentrations. (C) Conductance responses of bare, 1-pyrenebutanol-modified, and CCD1-modified CNT-FETs to Cys (1 aM to 10 μM). (D) Selective responses of CCD-modified CNT-FETs to Cys, GSH, Hcy, Met, Trp and Phe.

Taking advantage of the selective chemodosimetric reaction of CCD1 on the CNT-FET device, we demonstrated highly selective and ultrasensitive FET-based analysis of Cys in real time, as a model analyte, which is one of the essential amino acids. Abnormal levels of Cys are associated with several health problems, such as slow growth of children, hair depigmentation, edema, lethargy, oxidative damage, liver damage, loss of muscle and fat, skin lesions and weakness, metabolic disorders, and AIDS. We performed extensive control experiments to examine the effect of Cys on a CNT-FET modified with CCD1. To choose the optimal conditions for Cys detection based on the CCD1-modified CNT-FET, some key factors such as pH and immobilization time for CCD1 on CNT surface were examined. As shown in Fig. S3,† the buffer solution at pH 7.4 was optimal for detection Cys, and 60 min was enough for the immobilization of CCD1 on CNT-FET. Under the optimized experimental conditions, our sensing strategy was demonstrated by testing Cys of various concentrations. First, a 9 μL droplet of PBS (10 mM, pH 7.4) was placed on the CCD1-modified CNT-FET. The source–drain current was then monitored after the introduction of a Cys solution. A 0.1 V bias voltage was maintained at all times during electrical measurement. Fig. 3A shows the time dependence of the source-drain current for the CNT-FET sensor following successive addition of Cys. As we expected earlier from Fig. 1D, the addition of Cys caused a conductance change, *i.e.*, a sharp decrease in the source-drain current and then a gradual saturation at lower values. This indicates that with the addition of Cys, a gradual saturation was caused by the chemodosimetric reaction between CCD1 and Cys. Interestingly, even with a lower sensitivity, CNT-FET modified with CCD2 also showed electrical sensing behavior toward Cys molecules (Fig. S4†). This is presumably due to the highly sensitive nature of swCNTs to the changes in their chemical environment.^25,26^Fig. 3B shows the calibration curve of the CCD1-modified CNT-FET according to the Cys concentration, which exhibits a wide linear range (1 fM to 1 nM) over which there were measurable responses and a sensitive linear range with a slope of ∼0.062/decade. CCD2-modified CNT-FET also shows similar linear range, but ∼4 times less sensitive slope (∼0.015/decade) than CCD1-modified CNT-FET. The limit of detection (LOD) for CCD1-modified CNT-FET was estimated to be 0.45 fM (S/N = 3), which indicates ultra-sensitivity of our sensor compared to previous Cys sensors (Table 1). When we carried out the same measurements with bare CNT-FET and 1-pyrenebutanol-modified CNT-FET devices without CCD1 sensing motifs, the electrical conductance of the device exhibited almost no change upon the addition of Cys from 1 aM to 10 μM (Fig. 3C). This clearly shows that Cys sensing performance of the CCD1-modified CNT-FETs is mainly due to the Cys-selective chemodosimetric behavior of CCD.

**Table 1:** Comparison of various methods for the detection of Cys

Method	Materials	Linear range	LOD	Ref.
Colorimetry	Ag NPs	25–250 μM	20.63 μM	27
Colorimetry	Au NR	10 nM to 1 μM	10 pM	28
Colorimetry	Natural cellulose		20 μM	29
HPLC		1–20 μM	1 μM	30
Fluorescence	Ag cluster	25 nM to 6 μM	20 nM	31
Electrochemistry	Pt–CNT	500 nM to 100 μM	300 nM	32
Electrochemistry	SG-PEDOT/AuNP	0.1–382 μM	20 nM	33
This work	CCD1/CNT-FET	1 fM to 1 nM	0.45 fM	

The selectivity of our sensor toward interfering molecules was evaluated by extensive control experiments using various biologically relevant analytes including GSH, Hcy, Met, Trp and Phe. Fig. 3D shows conductance responses of the FET sensor based on CCD1-modified swCNTs upon the successive addition of Cys and potential interferences. All electrical conductance measurements exhibited negligible response upon the addition of control analytes, except Cys, revealing the exceptional selectivity of the sensor. We also demonstrated the application of the proposed assay system to human urine samples of healthy volunteers (Table 2). The results showed good recovery values, suggesting the suitability of the CCD1-modified CNT-FET sensor for the practical analysis of Cys in real samples. These analytical features of the modified CNT-based FET platform clearly demonstrate the role of CCD1 in the facile modification of CNT-based FET platforms for sensitive and selective FET-based analyses.

**Table 2:** Cys detection in human urine sample for real sample application of CCD1-modified CNT-FET sensor

Sample	Amount added (M)	Amount found (M)	RSD (%)	Recovery^a^ (%)
Urine	1.00 × 10^−14^	9.78 × 10^−15^	0.7	97.8
	1.00 × 10^−11^	1.00 × 10^−11^	0.6	100
	1.00 × 10^−9^	1.00 × 10^−9^	1.2	102

a Recovery (%) = (amount of found analyte/amount of added analyte) × 100.

## 4. Conclusions

In this work, the utility of the chemodosimetric sensing principle was demonstrated for the first time in the electronic biosensing with CNT-FET devices. The chemodosimeter-modified CNT-FET sensor exhibited the ultrasensitive detection of specific amino acids with an exceptional selectivity in real time. Such a high selectivity was realized using chemodosimeters as a sensing element, while CNT-FET allowed high sensitivity. It should be noted that chemical conformation caused by the chemodosimetric reaction can significantly alter the charge distribution on CNT surface, and thus excite CNT-FET conductivity responses. This strategy should allow us to overcome the poor selectivity problems of CNT-based FET sensors, and provides a powerful platform for the development of high-performance nano-biosensors.

## Conflicts of interest

There are no conflicts to declare.

## Supplementary Material

RA-009-C9RA04656A-s001
