# Supplementary material for: A chemodosimeter-modified carbon nanotube-field effect transistor: toward a highly selective and sensitive electrical sensing platform
Source: RSC Adv. 2019 Sep 9;9(49):28414–20. doi: 10.1039/c9ra04656a (PMC9071199; doi:10.1039/c9ra04656a)
Supplement: RA-009-C9RA04656A-s001 [file RA-009-C9RA04656A-s001.pdf]

Electronic Supplementary Material (ESI) for RSC Advances.
This journal is © The Royal Society of Chemistry 2019

Supporting information

A Chemodosimeter-Modified Carbon Nanotube-Field Effect Transistor: Toward Highly Selective and Sensitive Electrical Sensing Platform

Chang-Seuk Lee,[a] Jong Seung Kim,[b,*] and Tae Hyun Kim[a,*]

[a] *Department of Chemistry, Soonchunhyang University, Republic of Korea*

[b] *Department of Chemistry, Korea University, Republic of Korea*

**Corresponding authors. Tel.: +82-41-530-4722 (T. H. Kim), +82-2-3290-3183 (J. S. Kim); E-mail: thkim@sch.ac.kr (T. H. Kim), jonskim@ khu.ac.kr (J. S. Kim)*

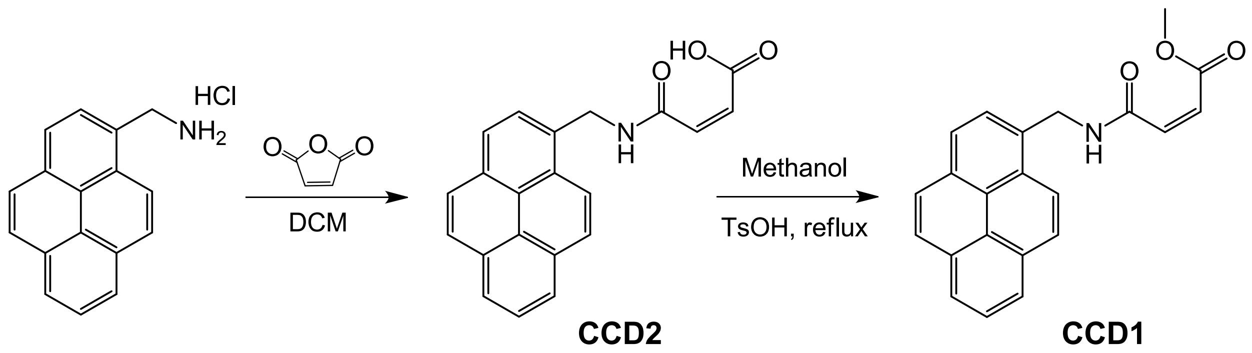

Scheme. S1. Synthesis mechanism of the pyrene based cysteine selective chemodosimeter (CCD).

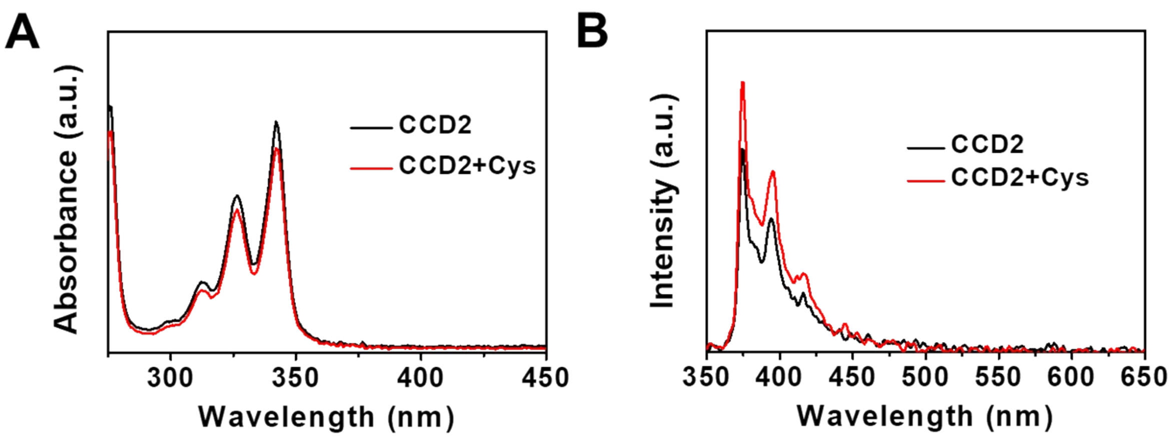

Fig. S1. (A) UV-vis and (B) fluorescence spectra of CCD2 (5.0 μM) in aqueous solution (10 mM PBS buffer, pH 7.4, 10% DMSO) upon addition of Cys (100 equiv). Excitation at 343 nm after 10 min (slit=1.5/3).

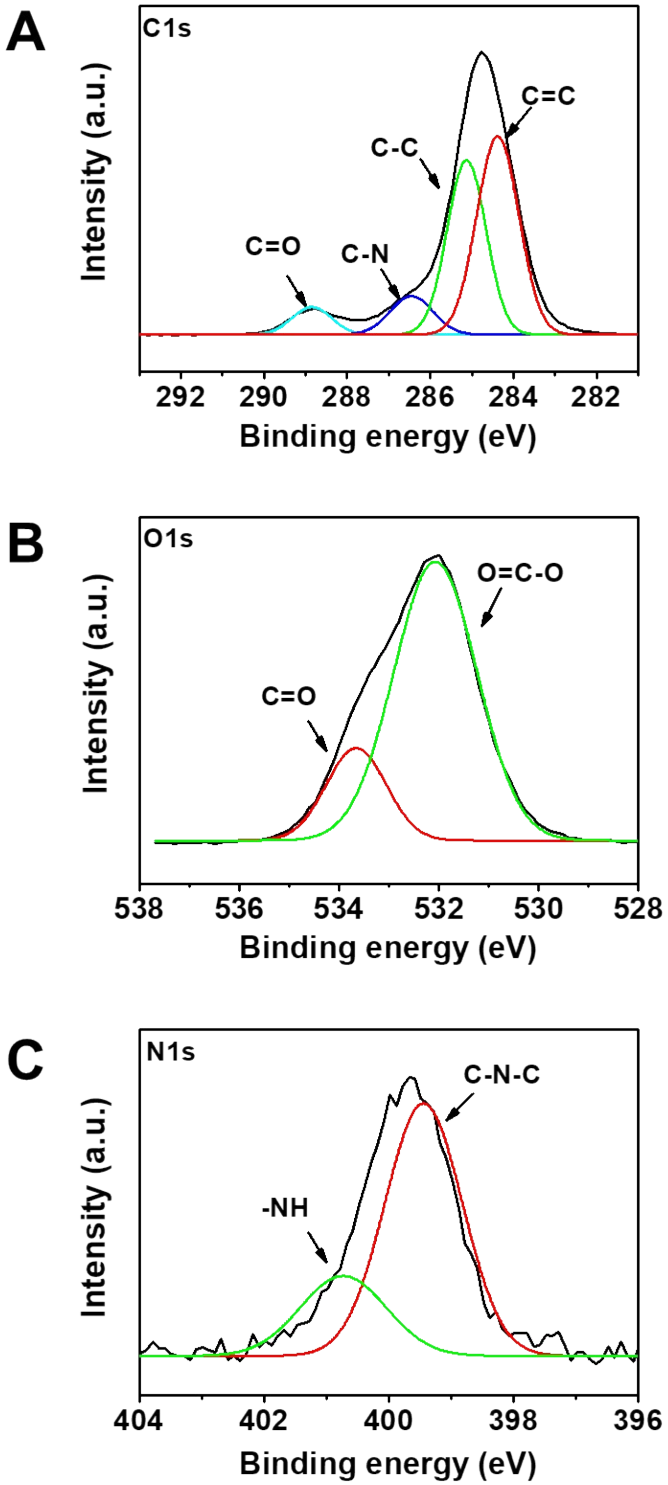

Fig. S2. (A) C 1s spectrum, (B) O 1s spectrum, and (C) N 1s spectrum of XPS analysis obtained in CCD1 functionalized swCNTs.

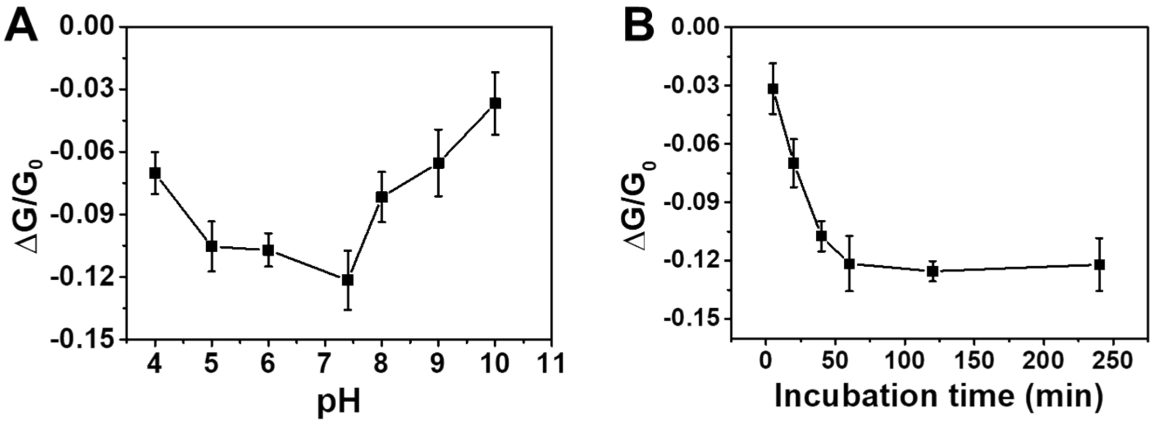

Fig. S3. The optimization of detection conditions. Effects of (A) the pH of the reaction solution and (B) the incubation time for CCD1 immobilization on the CNT-FET conductance response. The injected Cys concentration was 10 μM, and the 0.1 V was applied to CCD1-modified CNT-FETs for conductance measurement.

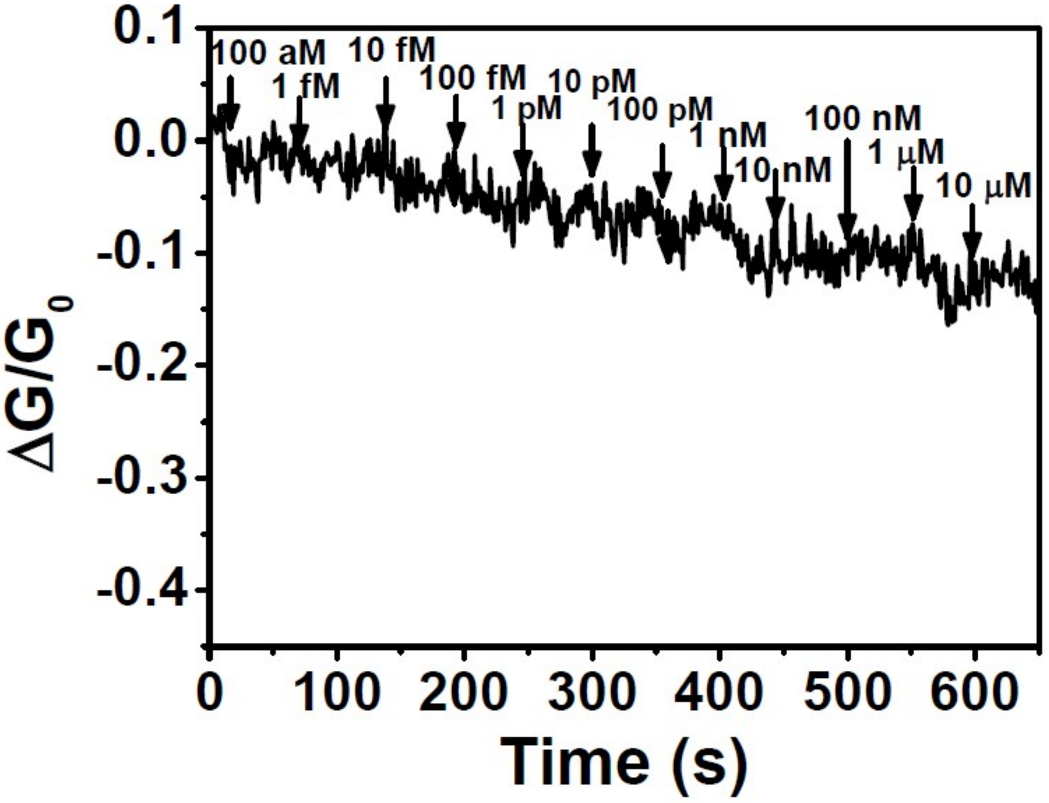

Fig. S4. Real-time conductance measurement data obtained from CCD2-modified CNT-FETs upon successive addition of Cys at various concentrations. Arrows indicate the injection points of Cys target molecules.
